# Fatality from COVID-19 does not affect palliative care duration among patients with advanced cancer: a retrospective cohort study

**DOI:** 10.31744/einstein_journal/2024AO0536

**Published:** 2024-09-25

**Authors:** Rafael Tavares Jomar, Jéssica Thaís Nascimento Marques, Livia Costa de Oliveira, Gelcio Luiz Quintella Mendes, Daianny Arrais de Oliveira da Cunha, Raphael Mendonça Guimarães

**Affiliations:** 1 Instituto Nacional de Câncer Rio de Janeiro RJ Brazil Instituto Nacional de Câncer (INCA), Rio de Janeiro, RJ, Brazil.; 2 Fundação Oswaldo Cruz Escola Nacional de Saúde Pública Sergio Arouca Rio de Janeiro RJ Brazil Escola Nacional de Saúde Pública Sergio Arouca, Fundação Oswaldo Cruz, Rio de Janeiro, RJ, Brazil.

**Keywords:** COVID-19, Neoplasms, Underlying cause of death, Palliative care, Pandemics, Survival, Cause of death

## Abstract

**Objective:**

This study aimed at investigating the extent to which COVID-19-induced fatalities affect the duration of palliative care among patients with advanced cancer.

**Methods:**

A retrospective cohort study was conducted at the Palliative Care Unit of the Brazilian *Instituto Nacional de Câncer* in Rio de Janeiro, Brazil, on 1,104 advanced cancer patients who died under exclusive palliative care between March 11, 2020, and March 31, 2021. Wilcoxon rank-sum (Mann-Whitney U) and log-rank tests were performed to examine statistical differences between the medians of time, and the Kaplan-Meier estimator was used to graphically illustrate survival over time under exclusive palliative care contingent upon the underlying causes of death of the two experimental groups (cancer *versus* COVID-19).

**Results:**

A total of 133 (12.05%) patients succumbed to COVID-19. In both groups, the median time under exclusive palliative care was less than one month. The exclusive palliative care survival curves did not exhibit any statistically significant difference between the groups.

**Conclusion:**

Death due to COVID-19 did not modify the duration of exclusive palliative care among patients with advanced cancer.

## In Brief

Jomar et al. demonstrated that death due to COVID-19 did not affect the time under exclusive palliative care among patients with advanced cancer, even during the first year of the pandemic caused by a hitherto little-known disease.

## Highlights

◼ Fatality due to COVID-19 does not alter the time under oncological palliative care.
◼ The retrospective design of this pioneering study allows causal inference.
◼ Access to oncological palliative care frequently approaches terminality of life.

## Graphical abstract

**Figure:**
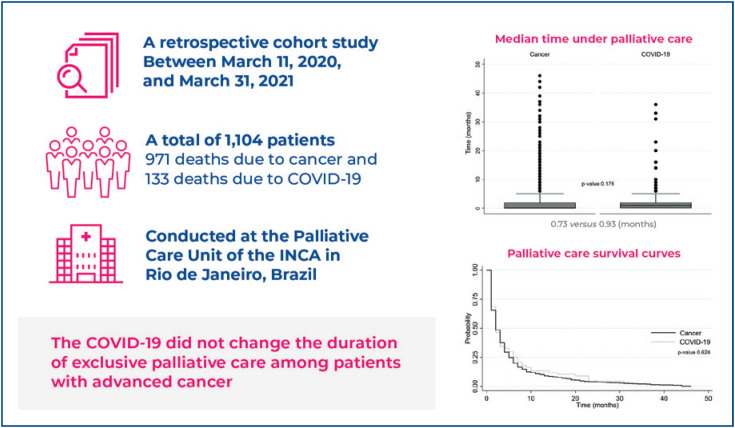

## INTRODUCTION

The emergence of the COVID-19 pandemic triggered by SARS-CoV-2 has induced a substantial alteration in the mortality profiles and healthcare provisions for cancer patients worldwide. The year 2020 witnessed approximately one million additional deaths in 29 high-income countries compared to the previous four years.^(1)^ Moreover, delays and disruptions plagued the provision of cancer services, primarily affecting facilities (up to 77.5%), supply chains (up to 79%), and personnel availability (up to 60%), particularly in Europe and North America.^(2)^

In 2020, COVID-19 ranked as the third most prevalent cause of death in Brazil, with reduced cancer-related mortality as the underlying cause (−9.71%) and increased mortality from cancer as a contributory cause (+82.05%) compared to 2019.^(3)^ Therefore, COVID-19, as a competing cause of death, resulted in the shifting of the underlying cause of death, where the prevalent cases of cancer would have a higher risk of death from this disease than anticipated due to COVID-19.^(4)^ Regarding the screening, diagnosis, and treatment of cancer in Brazil in 2020, a reduction in cervical cytology tests (−44.6%), mammograms (−42.6%), biopsies (−35.3%), anatomopathological exams (−26.7%), cervical excisions (−32.6%), surgeries (−15.7%), and radiotherapy procedures (−0.7%) was noted compared to 2019.^(5)^

Although extant literature delineates the effects of the pandemic on the screening, diagnostic investigation, and treatment of cancer, data on patients under exclusive palliative care due to advanced cancer during this period, such as pandemic-related mental crises, additional symptom burden post-COVID-19, and the accessibility of palliative care services, remains scarce.^(6)^ Therefore, this study hypothesizes that COVID-19-associated fatalities may decrease the time under palliative care among patients with advanced cancer.

## OBJECTIVE

To investigate the extent to which COVID-19-induced deaths affect the duration of palliative care among patients with advanced cancer.

## METHODS

The current retrospective cohort study was conducted at the Palliative Care Unit of the Brazilian *Instituto Nacional de Câncer* in Rio de Janeiro, Brazil, which provides medical care to manage symptoms and enhance the quality of life of patients with advanced cancer. All patients considered for this study were admitted into either one of four Brazilian *Instituto Nacional de Câncer* hospital units after exhausting all therapeutic possibilities without any substantial result. As their conditions were incurable, they received specialized palliative care both in the hospital and at home instead of undergoing any antineoplastic treatment with control intent.

The data for the study were sourced from the death certificates of all advanced cancer patients who died at the palliative care units during the COVID-19 pandemic,^(7)^ between March 11, 2020, and March 31, 2021. The hospital administration issued death certificates, from which we collected the following data: date of death, age (less than or equal to the median *versus* greater than the median), sex (male *versus* female), race/complexion (white *versus* non-white), level of schooling (none or elementary school *versus* middle school *versus* high school or more), marital status (single, divorced, or widowed *versus* married or stable union), city of residence (Rio de Janeiro *versus* others), place of death (hospital *versus* other health facilities, home or other places), underlying cause of death (cancer *versus* COVID-19), and contributory cause of death (cancer subtypes).

Additionally, the electronic health records of the patients were consulted to extract the admission date to the palliative care unit. We obtained the time under exclusive palliative care (in months) by calculating the difference between the admission date to the palliative care unit and the date of death. At this stage, three patients lacking documented admission dates were excluded from the study (one of them evidently died from COVID-19). The data were collected between August and November 2021.

The pandemic prompted us to adhere to the international standard proposed by the World Health Organization to certify the causes of death, which stipulates that comorbidities, such as cancer, should not be considered as an underlying cause of death despite aggravating the pathogenesis of COVID-19.^(8)^ Therefore, we designated cancer subtypes and COVID-19 as the contributory and underlying causes of death, respectively, when both causes were documented on the death certificate.

Sociodemographic characteristics were delineated using proportions, and Pearson's χ^2^ or Fisher's exact tests were used to examine statistical differences between the causes of death among the groups. Wilcoxon rank-sum (Mann-Whitney U) and log-rank tests were performed to evaluate statistical differences between the medians of time, and the Kaplan-Meier estimator was used to graphically illustrate survival over time under exclusive palliative care contingent upon the underlying cause of death of the two experimental groups (cancer *versus* COVID-19). All statistical analyses were performed using Stata 15.0, with a statistical significance level of 0.05.

The *Instituto Nacional de Câncer* Ethics Committee waived the requirement for informed consent and approved this study on May 17, 2021 (CAAE: 46308721.7.0000.5274; #4.716.122).

## RESULTS

This study comprised a total sample size of 1,104 patients with advanced cancer (99.72% eligible). The mean and median ages of the patients were 62.08 (±13.56) and 62.50 years, respectively. There were no statistical differences in the sociodemographic characteristics between the groups (Table 1).

**Table 1:** Sociodemographic characteristics according to the cause of death

Variables*		Cause of death		p value
		Cancer n (%)	COVID-19 n (%)	
Age group (in years)				0.781†
	20–62	487 (50.15)	65 (48.87)	
	63–99	484 (49.85)	68 (51.13)	
Sex				0.540†
	Male	392 (40.37)	50 (37.59)	
	Female	579 (59.63)	83 (62.41)	
Race/skin color				0.374†
	White	421 (43.63)	63 (47.73)	
	Non-white	544 (56.37)	69 (52.27)	
Level of schooling				0.500†
	None or elementary school	313 (32.60)	40 (30.77)	
	Middle school	277 (28.85)	44 (33.85)	
	High school or more	370 (38.54)	46 (35.38)	
Marital status				0.088†
	Single, divorced, or widowed	569 (59.33)	68 (51.52)	
	Married or stable union	390 (40.67)	64 (48.48)	
City of residence				0.552†
	Rio de Janeiro	528 (54.83)	76 (57.58)	
	Others	435 (45.17)	56 (42.42)	
Place of death				0.499‡
	Hospital	948 (97.93)	132 (99.25)	
	Other health facilities, home, or other places	20 (2.07)	1 (0.75)	

* Missing information excluded from percentage calculations: race/skin color (n=7), level of schooling (n=14), marital status (n=13), city of residence (n=9), and place of death (n=3)

† p value refers to Pearson χ^2^ test

‡ p value refers to Fisher's exact test.

**Figure 1:**
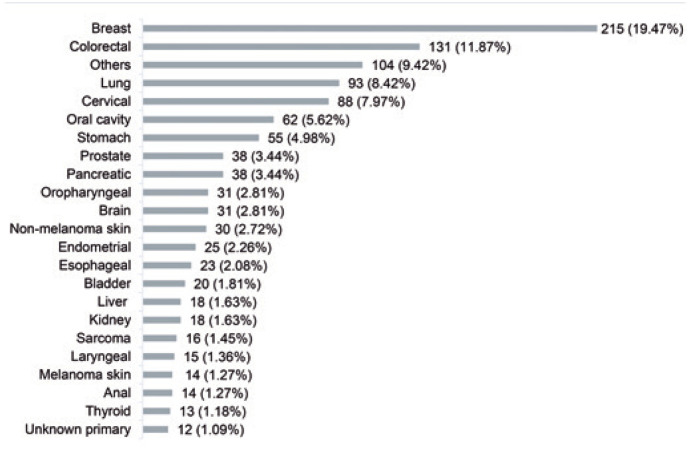
Frequency of cancer types that caused deaths, both as underlying and contributory causes

A total of 133 (12.05%) advanced cancer patients were reported to have died of COVID-19. The cancer types that served both as underlying and contributory causes of death are depicted in figure 1, with breast (19.47%) and colorectal cancers (11.87%) being the most frequent.

**Figure 2:**
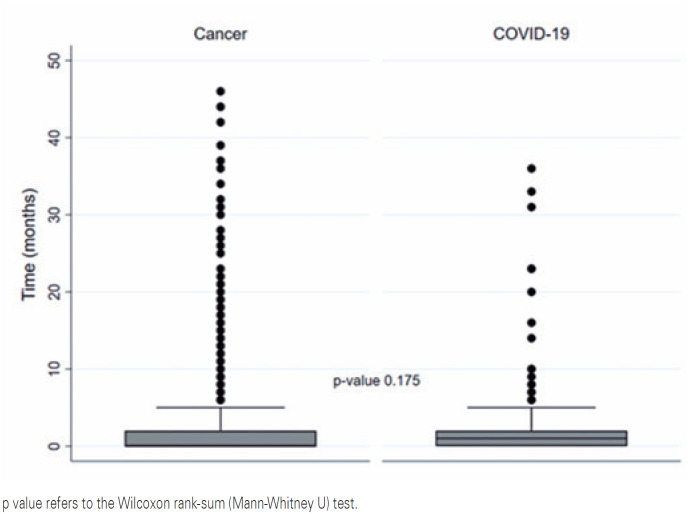
Boxplot of time under exclusive palliative care by cause of death

The mean and median times under exclusive palliative care were 2.37 (±5.77) and 0.73 months, respectively, and the maximum was 46 months. The median time under exclusive palliative care was less than one month in both groups of patients who died from either cancer or COVID-19 (Figure 2) and did not exhibit any statistical difference between the groups (respectively, 0.73 *versus* 0.93; p=0.175).

**Figure 3:**
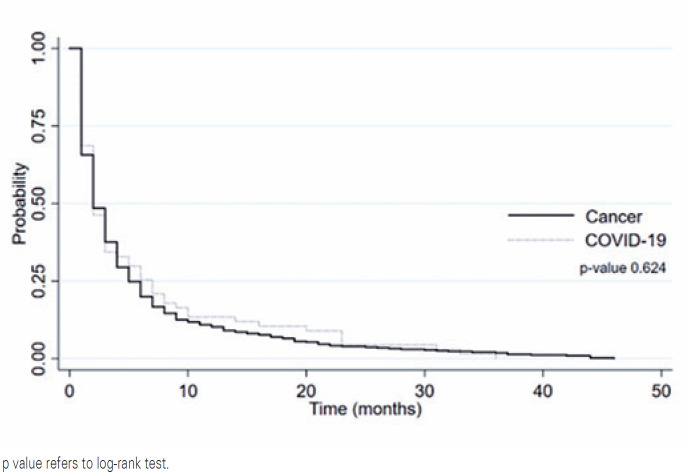
Exclusive palliative care survival curve according to cause of death

The exclusive palliative care survival curves illustrated in figure 3 did not portray statistical differences between the groups of patients who died of either cancer or COVID-19 (p=0.624).

Others (Figure 1) refer to the sum of cancer types with with less than 10 cases, namely: hypopharyngeal (n=8), sinus (n=8), ill-defined site (n=7), lynphoma (n=7), nasopharyngeal (n=6), bones/joints of other sites (n=5), meningioma (n=5), bone/joints of the limbs (n=4), gallbladder (n=4), hematopoietic/reticuloendothelial system (n=4), nasal cavity/middle ear (n=4), spinal cord/cranial nerves/other sites of the central nervous system (n=4), unspecified parts of the bile ducts (n=4), vulvar (n=4), eye (n=3), heart/mediastinum/pleural (n=3), ill-defined site of the lip/oral cavity/pharynx (n=3), renal pelvic (n=3), uterine (n=3), other digestive organs (n=2), parotid gland (n=2), retroperitoneal/peritoneal (n=2), salivary gland (n=2), small bowel (n=2), thymic (n=2), other female genital organs (n=1), penile (n=1), and tonsil (n=1).

## DISCUSSION

The study findings revealed that the duration of exclusive palliative care among patients with advanced cancer remained unaltered despite the escalation in COVID-induced mortality during the first year of the pandemic caused by this hitherto little-known disease. Notably, Brazil was among the countries that were most severely affected by COVID-19. Despite accounting for a mere 2.7% of the global population, as of March 31, 2021, Brazil has reported 12,753,258 and 321,886 COVID-19-related cases and deaths, respectively,^(9)^ which represent 10.1% and 11.6% of the worldwide instances of infection and fatalities,^(10)^ respectively. As of March 31, 2021, Rio de Janeiro had documented a total of 20,320 fatalities attributed to COVID-19, establishing it as the city with the highest COVID-19-associated death toll in the country.^(11)^

The brief median time under exclusive palliative care draws attention as it prompts the hypothesis that access to palliative care is frequently attained close to terminality and the finitude of the lives of the patients, as evidenced by studies conducted prior to the COVID-19 pandemic.^(12-17)^ A qualitative study documented that according to oncologists employed at the Brazilian *Instituto Nacional do Câncer* hospital units, referring patients to exclusive palliative care is complicated and may be hampered by challenges related to the profession, the expectations of patients and their families, and the characteristics of the institute, which contribute to the delayed admittance to said care.^(18)^ Prior to the COVID-19 pandemic, research conducted in an identical backdrop reported that patients with advanced cancer displayed a median overall survival between 39 (interquartile range: 26-90) and 53 (interquartile range: 20-90) day;^(19,20)^ conversely, international investigations presented a median time under exclusive palliative care for advanced cancer between 20 (interquartile range: 8-45) and 42 (interquartile range: 15-126) day.^(13,15,17)^

COVID-19-associated fatality arguably did not alter the time under exclusive palliative care owing to the fact that the patients were referred to the palliative care unit in close proximity to the terminality and finitude of life due to advanced cancer. Thus, implementing a patient-centered palliative care culture could gradually reverse this scenario of offering tumor-targeted therapy with no prospect of disease modification, which fails to effectively manage the most prevalent and distressing symptoms of cancer patients and, consequently, to improve their quality of life.^(18)^

Prior research has indicated that the COVID-19 pandemic did not threaten the survival of cancer patients under palliative care,^(21)^ but rather increased the death toll within 24 hours of admission.^(22)^ Furthermore, a higher risk of death was noted among COVID-19 cases in aged patients with advanced cancer under exclusive palliative care, with lung tumors (primary or metastases), and chronic obstructive pulmonary disease.^(23)^ To the best of our knowledge, this study is the first to substantiate that death due to COVID-19 does not impact the duration of exclusive palliative care among patients with advanced cancer.

This study was strengthened by certain aspects. First, it involved a large cohort of patients from a proficient palliative cancer care center. Second, its retrospective design allowed causal inference. Third, the data were analyzed using distinct and appropriate statistical procedures. Lastly, compliance with the international standard for certifying causes of death during the COVID-19 pandemic^(8)^ evidently minimized the occurrence of misclassification bias.

However, this study had certain limitations. First, although this study center is reputably the most prominent national reference center for facilitating palliative care for cancer patients through the Brazilian Public Health System, it constrains the sample source to a single center. Second, the data was primarily sourced from death certificates; this secondary data source hindered the evaluation of other important COVID-19-associated variables, including diagnostic confirmation by reverse transcription-polymerase chain reaction and precautionary measures, namely social isolation, mask use, and frequent hand washing during the pandemic. Considering that patients under palliative care and their caregivers may have exercised substantial caution and adhered to the aforementioned measures, the study results warrant thorough interpretation.

## CONCLUSION

In conclusion, conducting additional multicenter studies that encompass diverse data sources can further confirm to the lack of impact of COVID-19-related fatalities on the duration of exclusive palliative care among patients with advanced cancer, as evidenced by this particular study.
